# Dr. Abiah Morgan Cartledge

**Published:** 1908-06

**Authors:** 


Dr. Abiah Morgan Cartledge, of Louisville, Ky., died suddenly at his apartments in that city, May 4, 1908, of angina pectoris, aged 49 years. Though he had not been altogether in perfect health for the last few weeks of his life, death finally came as a great shock to the profession of Louisville and to the community at large. His wife died in September, 1907. He is survived by a daughter, Miss Emma Cartledge, sixteen years old, two sisters, Mrs. Laura J. Crockett, who had been living with

Dr. Cartledge since the death of his wife, and Mrs. A. B. Wilson, of Greenville, Tenn.

Dr. Cartledge was a native of North Carolina and came to Louisville at the age of eighteen, at once taking up the study of medicine and graduating from the Hospital College of Medicine in that city in 1887. After graduation he at once entered upon the general practice of medicine. Very soon, however, his aptitude for surgery became apparent and won for him enough of that work to fully occupy his time. It was not long before he limited his professional practice entirely to surgery, becoming, while yet in his early professional career, one of the famous surgeons of the South.

Dr. Cartledge was a member of the American Medical Association; of the Southern Surgical and Gynecological Association (president in 1900) ; professor of abdominal surgery and gynecology in the Louisville Medical College; surgeon to the Louisville, Saint Louis & Texas Railroad, and to the Louisville City Hospital.

Dr. Cartledge presented a striking figure, being a handsome man in the best sense of the term. He was a most companionable person, a representative of the best type of clubman, a bon vivant and a raconteur, possessing special gifts on both these lines. He was amiable, self-poised, a delightful companion and a splendid citizen. Peace to his ashes.

